# A 750 K Photocharge Linear Full Well in a 3.2 μm HDR Pixel with Complementary Carrier Collection †

**DOI:** 10.3390/s18010305

**Published:** 2018-01-20

**Authors:** Frédéric Lalanne, Pierre Malinge, Didier Hérault, Clémence Jamin-Mornet, Nicolas Virollet

**Affiliations:** STMicroelectronics. 850 rue Jean Monnet, 38926 Crolles Cedex. France; pierre.malinge@st.com (P.M.); didier.herault@st.com (D.H.); clemence.jamin@st.com (C.J.-M.); nicolas.virollet@st.com (N.V.)

**Keywords:** HDR imaging, CMOS image sensors, outdoor vision

## Abstract

Mainly driven by automotive applications, there is an increasing interest in image sensors combining a high dynamic range (HDR) and immunity to the flicker issue. The native HDR pixel concept based on a parallel electron and hole collection for, respectively, a low signal level and a high signal level is particularly well-suited for this performance challenge. The theoretical performance of this pixel is modeled and compared to alternative HDR pixel architectures. This concept is proven with the fabrication of a 3.2 μm pixel in a back-side illuminated (BSI) process including capacitive deep trench isolation (CDTI). The electron-based image uses a standard 4T architecture with a pinned diode and provides state-of-the-art low-light performance, which is not altered by the pixel modifications introduced for the hole collection. The hole-based image reaches 750 kh+ linear storage capability thanks to a 73 fF CDTI capacitor. Both images are taken from the same integration window, so the HDR reconstruction is not only immune to the flicker issue but also to motion artifacts.

## 1. Introduction

A realistic scene for an automotive camera may include fast-moving objects, pulse-width-modulated light sources, and a very high dynamic background over 130 dB. Such a scene requires the image sensor to be highly sensitive with high dynamic range capability and immunity to the flicker effect.

Several methods of implementing high dynamic range (HDR) capabilities in a CMOS (Complementary Metal Oxide Semiconductor) image sensor have been developed thus far. They are listed below with their main advantages and draw-backs.

### 1.1. Staggered HDR

The most straightforward approach is the staggered HDR [1] with a consecutive integration of a long and a short image. In this case, no change of pixel architecture is needed, the two images are managed by the video timing, and a partial frame buffer memory is needed to store image rows in between a long and a short readout. The main weakness here is due to the second exposure, which can be shorter than the period of a pulse-width-modulated light source generating the well-known flicker effect. Staggered HDR imaging may be very flexible, with a variable integration time ratio between long and short exposure and the addition of even shorter images to increase the overall dynamic range.

### 1.2. Chopped HDR

To address the flicker issue, the short image can be chopped into multiple time slots [2]. At pixel level, the addition of a reset gate to drain the photodiode between exposure sub-windows is required. A short image signal is then accumulated on the floating diffusion node with an iteration of transfer gate pulses. This chopper HDR solution mitigates the flicker issue. However, it does not solve the motion artifact effect, keep the need of image buffer memory, and degrades the frame rate.

### 1.3. Lin-Log Pixel

The lin-log HDR technique leverages the typical dual regime of pinned photodiodes, switching to a logarithmic response when the signal is above a standard full well. Unfortunately, extremely high fixed pattern noise (FPN) is reported for the logarithmic part of the curve [3,4]. This FPN can be reduced with a pixel-by-pixel offset calibration [3], and the offset can be extracted at each row with a specific pixel readout timing after the signal readout. Even after calibration, the FPN may remain significantly high. Finally, although this solution is based on a single image, it cannot be considered as flicker-free. Indeed, the HDR behavior is due to the photodiode overflow current, which is an exponential function of the amount of charges present in the photodiode (Qpd). For continuous light at a high signal, the electron flux is equal to the overflow current and can be computed from the Qpd readout. For a pulsed modulated source, during the light pulse the Qpd will settle to the level associated with the electron flux, but in between light pulses the charges’ quantity above the linear full well level will be drained rapidly. Since the light-emitting diode (LED) pulses may occur randomly versus pixel readout, the output signal will be strongly variable and will generate blinking images.

### 1.4. Dual-Diode Pixel

To address both the flicker and motion artifact issues, a first solution can be the introduction of a second photodiode inside the pixel [5]. Optimized for a high signal level, this second photodiode should have the highest possible full well and lowest possible sensitivity, whereas the noise performance target can be significantly relaxed as long as it remains below the photon shot noise level. Beside the complexity of the realization of such pixel, the main drawback of the dual-diode HDR is the loss of quantum efficiency of the main diode, since the incoming signal is split between the two diodes. A minimum of 5% relative degradation must be expected. Combining a dual diode and the chopped HDR concept enables an extremely flexible HDR solution [6], enabling an extended dynamic range while keeping strong immunity to the flicker effect.

### 1.5. Lateral Overflow Pixel

A smart simplification of the dual photodiode concept leads to the lateral overflow (LOF) architecture [7]. The pixel works with a single photodiode, but keeps two storage areas: the diode and an added capacitor, which stores the overflowing current as soon as the diode is saturated. Two sequential readouts provide the diode-only signal first and the combined diode and overflow signal in a second step. In this case, the pixel design is similar to a dual conversion gain (CG) except the addition of one passive element: the overflow capacitor. The dynamic range offered with the LOF pixel is directly linked to the ability to integrate very high value capacitor in the pixel area, most likely in the back-end for back-side illuminated (BSI) sensors. As an example, a 375 ke− full well has been reported in a 6 × 6 µm pixel [8].

### 1.6. Complementary Carrier Pixel

The complementary carrier collection pixel concept has been disclosed at the 2017 International Image Sensor Workshop held in Hiroshima [9]. Its architecture and implementation results are reproduced hereafter. At first glance, the approach is similar to the lateral overflow technique with a single photodiode and a capacitor storing the overflow signal. The fundamental difference is in the inverted polarity solution for high signal management. This architecture directly addresses the main difficulty of the LOF pixel with the easy integration of a high-value capacitor thanks to the capacitive deep trench isolation (CDTI) process. Furthermore, it offers a higher voltage swing, which can be converted in enhanced dynamic range or in a higher signal-to-noise ratio (SNR). The only draw-back is the need for an additional transistor (RST_PSUB) and for P-type well (PWELL) isolation, which may limit the ability to shrink this pixel.

Table 1 summarizes the PRO and CONS for the above-described approaches. In the following, we will describe the complementary carrier pixel architecture and compare its theoretical SNR performance to the alternative solutions listed in this table, which are also immune to the flicker effect.

## 2. Complementary Carrier Pixel Introduction

### 2.1. Electron Imaging

**Figure 1:**
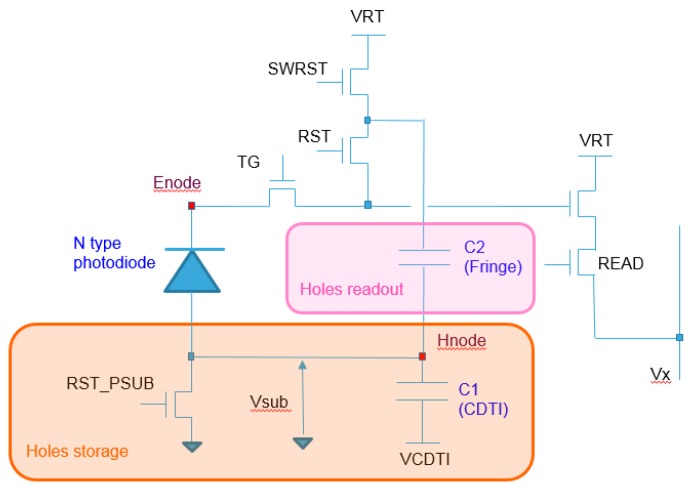
Pixel schematic. TG: transfer gate; RST: reset transistor in high CG mode; CDTI: capacitive deep trench isolation; VRT: positive supply voltage; SWRST: reset transistor in low CG mode; VCDTI: CDTI biasing voltage.

In this pixel, electron collection, storage, and readout are performed in a similar manner to the standard 4T operations. In the schematic (Figure 1), the non-highlighted part represents the electron circuitry with a dual CG 5T architecture. Such an arrangement allows for a single-exposure dual CG true CDS (correlated double sampling) readout with a seamless connection between modes [10,11]. Thus, more than 25 ke− can be read with perfect quality thanks to the 160 µV/e− high-conversion gain associated with the 30 µV/e− low-conversion gain and the 800 mV linear swing on the column.

### 2.2. Hole Collection and Storage

With a typical 4T-style pixel, once an electron–hole pair has been generated by an incident photon, the electron is collected, but the hole—which is considered to be redundant—is drained through the substrate contact and lost. For the complementary carrier pixel, in order to permit hole collection, the substrate of each pixel is electrically isolated with respect to the neighboring ones. This isolation is achieved through the use of deep trenches, which penetrate the full silicon depth of the BSI sensor and fully surround the photodiode area. Such deep silicon trenches have already been used to prevent electrical crosstalk in BSI sensors [12]. As shown in Figure 1, an N-type transistor (RST_PSUB) is used to reset the pixel substrate to ground at the beginning of the integration or during the readout phase.

All the transistors except the transfer gate are located in between two photodiodes with a P-well connected to ground. This constraint reduces the photosensitive area.

Once the photodiode is saturated, the electrons are drained through the transfer gate. It is important that 100% of the electrons are extracted in order to avoid recombination. A too-high transfer gate barrier may degrade electron overflow and impact hole signal uniformity or linearity (in case the barrier level depends on the Vsub bias).

The HDR performance of this pixel architecture is directly linked to the capability to store holes in quantities greatly exceeding the electron full well capacity. The hole full well is a function of the hole node capacitance (Chn): a 73 fF capacitance will store 912 kh+ over a 2 V swing. This capacitance value is mostly achieved by utilizing the isolation trenches (C1). The C2 fringe capacitor, transfer gate, and other second-order parasitic capacitances also contribute to Chn. Isolation trenches forming C1 are fabricated with an oxide liner and subsequently filled with polysilicon to form capacitive deep trench isolation (CDTI) [13]. Since CDTI is used for both diode isolation and the hole storage capacitor, there is no design flexibility to tune the C1 value.

**Figure 2:**
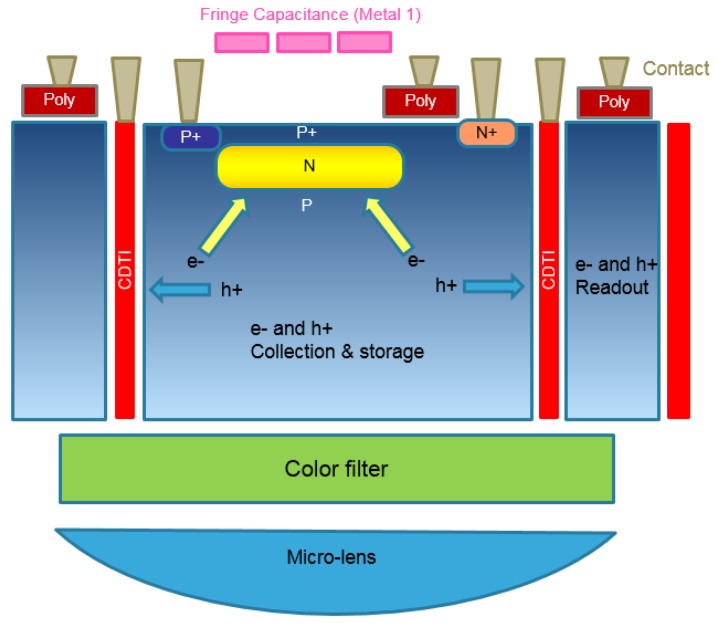
Schematic cross-section of the pixel.

A schematic cross-section view of such a pixel is presented in Figure 2.

### 2.3. Hole Readout

The hole signal is the voltage reached by the pixel substrate (Vsub). Starting from 0 V, and with a potentially large swing, it is not practical to directly measure this with an NMOS transistor, therefore a capacitive-coupling design has been chosen instead. The pixel substrate is coupled to the sense node through C2, which includes a fringe capacitor and the transfer gate’s drain junction.

$$\mathrm{Hole}\;\mathrm{read}\;\mathrm{out}\;\mathrm{signal}=\frac{q\cdot \left(\mathrm{hole}\;\mathrm{number}\right)}{\mathrm{Chn}}\cdot \frac{C2}{\mathrm{Csn}}\cdot \left(\mathrm{SF}\;\mathrm{gain}\right)$$

Unlike for electrons, only one CG is available for hole readout.

The hole readout design has multiple benefits: firstly, it efficiently reuses the existing pixel circuitry; the only active device added to the pixel is the RST_PSUB transistor. Secondly, it offers ample flexibility of the capacitance ratio C2/Csn (sense node capacitance) to enable the high-voltage swing on Vsub to be converted to a lower swing acceptable for the readout chain. Finally, it must be noted that although the hole signal is positive, the 3T-like timing with the signal sampling preceding the reset sampling produces—like the electron signal readout—a negative voltage swing at the column, hence the same analog-to-digital conversion (ADC) scheme can be used for both electrons and holes.

As for any 3T readout scheme, the kTC noise must be considered. Given the extremely large hole storage capacitance and the C2/Csn coupling ratio, its contribution is not as dominant as might initially be expected. Indeed, the kTC noise for Chn = 73 fF with a 68% C2/Csn ratio is calculated to be 215 µV (Source follower gain 0.89, temperature 335 K), which is within the expected range of typical source follower noise or 12 bit ADC quantization noise.

The pixel schematic is illustrated in Figure 1. The pixel includes a nominal N-type photodiode, six transistors and two built-in capacitances. The transfer gate (TG), reset transistors (RST and SWRST), the source follower (SF), and READ transistors enable electron operation with two conversion gains (high and low). C1 is the CDTI capacitance used to store holes during integration, and C2 is the fringe capacitance coupling the Hnode to the sense node for hole readout. C1 and C2 do not negatively impact the photodiode fill factor: C1 is at the pixel edges and also serves as the pixel isolation; we take advantage of the BSI process in order to implement C2 in the back-end layers above the photodiode.

## 3. Readout Interference Considerations

**Figure 3:**
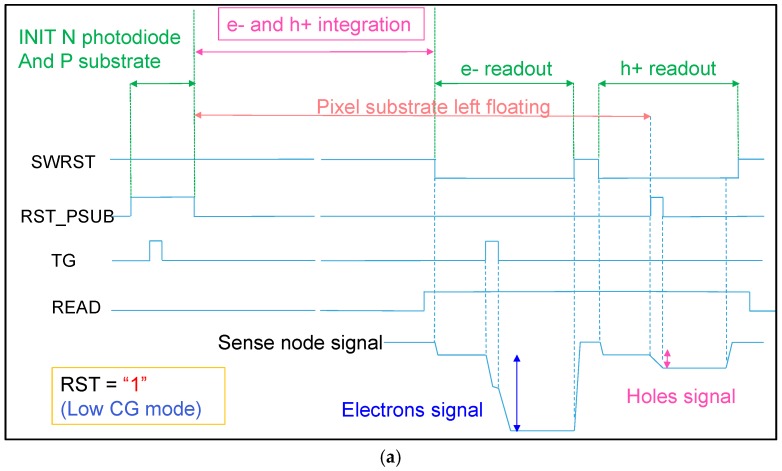

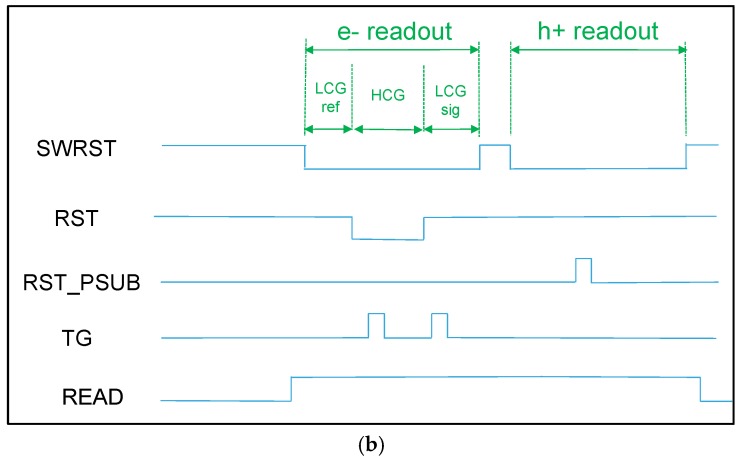
Pixel timing. (**a**) Full timing for low conversion gain mode (LCG); (**b**) readout timing with double conversion gain mode for electron. HCG: high conversion gain.

The pixel timing for low CG gain is presented in Figure 3a. After an initialization period for cleaning the photodiode and the substrate, TG and RST_PSUB are turned off, which enables the start of electron and hole integration. During this integration, the CDTI capacitance C1 is used to store the holes in the substrate while limiting the substrate voltage increase.

At the end of the integration period, the N photodiode will have collected a number of electrons and the substrate voltage will have increased by up to +Vsub. The readout sequence starts with the readout of the electrons, followed by the readout of the holes. When RST_PSUB is asserted, Hnode is reset to Vss and the voltage variation –Vsub is injected to the sense node voltage through the capacitance C2. For HDR image reconstruction, we combine the electron and hole signal data for each pixel. Figure 3b describes the enhanced timing for this pixel with triple readout, starting with dual CG for the electron followed by hole readout. Such timing could be used to maintain the best possible SNR along the whole dynamic range.

Compared to a standard 4T pixel, the electron readout slightly differs in that the diode is embedded in a floating pixel substrate and the sense node is coupled to this same floating node (Hnode). For signals significantly above the electron full well, Hnode rises towards a high voltage and can potentially impact electron readout. However, in these circumstances the electron data is irrelevant, since the pixel information is provided by holes anyway. For low signals, Hnode is always close to 0 V, and due to the strong coupling to the CDTI trench, the only likely artefact to be found is a harmless increase of the conversion factor.

While the hole signal has a limited effect on the electron readout, the converse is not true. Electrons in the photodiode can directly impact the hole readout and the decision on which charge carrier to extract first must be carefully considered. Indeed, hole-first timing shows no sensitivity at a low signal until the diode reaches its full capacity. The generated holes are stored in the pinned-junction-embedded capacitance to balance their electron counterpart. Hence, Vsub does not rise until the diode becomes full and the additional electrons start to overflow through the transfer gate channel. As a consequence, the image reconstruction is the sum of the hole and the electron signals. This also implies that all of the electrons stored in the photodiode are accurately counted, which may not be always true, for example, when high conversion gain is used.

Because of the issues highlighted with hole-first readout, we consider electron-first timing as a more robust approach. During the electron readout phase, the photodiode is reset and the hole signal is restored on Hnode. The subsequent hole readout is then fully linear from the first photon. At the other side of the curve, this electron-first approach may induce a slight non-linearity when Hnode approaches VRT (positive supply voltage). At some point, the pinning voltage of the photodiode will be higher than VRT and the reset will not be completed. For each electron kept in the diode, there will be a corresponding hole missing in the readout. This effect will progressively increase at very high signal levels, but it is only expected to reach a maximum of 3% non-linearity and should therefore not be detrimental to image quality.

**Figure 4:**
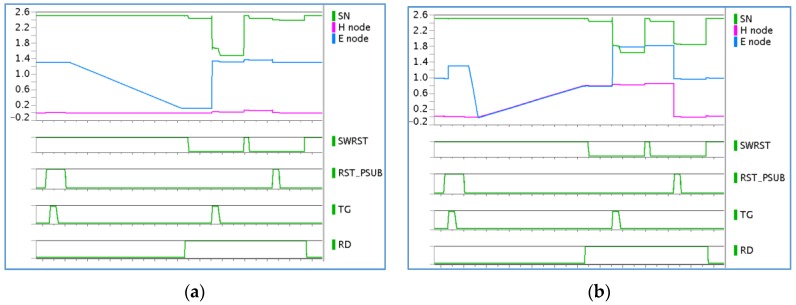
Pixel simulation. Signal: 30 k (**a**); 400 k (**b**). SN: Sense node.

Simulated Enode and Hnode behaviors for various illumination conditions are plotted in Figure 4.

## 4. Modeling and Comparing SNR for Multiple HDR Solutions

In this paragraph, the theoretical performance of different pixel architectures is studied regardless of the fact that such pixels may or may not be realistically built. For simplification, fix pattern noise and dark current are also ignored. Low signal imaging performance is assumed to be similar for all pixels, and we focus on the high signal level model.

The method consists in sizing the elements of each pixel to fit exactly a 1 M e− equivalent full well dynamic range capability. For the dual photodiode, a 10 X sensitivity ratio is arbitrarily chosen. This implies a 100 ke− secondary diode for which a 3T architecture is most likely, and consequently ktc noise is added into the model. We also consider the case where the ktc noise of the secondary diode is cancelled. The lateral overflow pixel capacitance value is 180 fF, which would require a very challenging 20 fF/µm² MIM capacitor process for a 3 µm pixel. For the complementary carrier pixel, the C2/Csn coupling ratio is optimized at 36% to ensure a 2.5 V linear swing for the hole-collecting node.

**Figure 5:**
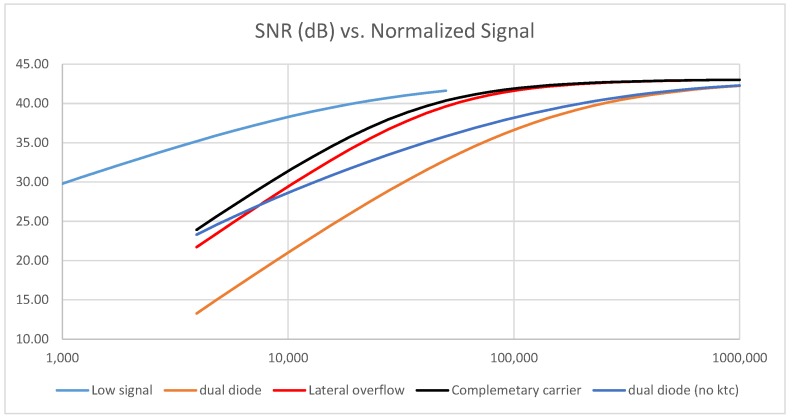
Architecture benchmark for a 1 Me− equivalent full well pixel, simplified model. SNR: signal-to-noise ratio.

All of the model inputs are listed in Table 2 as well as the resulting temporal noise. The signal-to-noise ratio as a function of normalized input signal is then computed by adding the shot noise contribution and 0.7% pixel response non-uniformity (PRNU). All of the above cases are plotted in Figure 5.

The graph demonstrates that in all cases, complementary carrier pixel architectures exhibit a better SNR than the other architectures, with a 2.3 dB advantage in the transition region versus the lateral overflow option. The difference is explained by the lower ktc noise thanks to a lower capacitance enabled by the wider voltage swing.

In the case of the dual diode concept, the 3× lower noise does not compensate for the 10× signal reduction, and leads to a 10 dB SNR gap. Cancelling the ktc noise is not effective enough to close that gap in the region of interest, highlighting that shot noise is also a strong SNR detractor for the dual diode.

Finally, this graph shows the potential for high-quality images in HDR mode with the complementary carrier technique. As an example, the SNR can be kept above 40 dB if the pixel includes a 50 ke− capable photo diode.

## 5. Results

### 5.1. SNR: Measurements versus Theory

**Figure 6:**
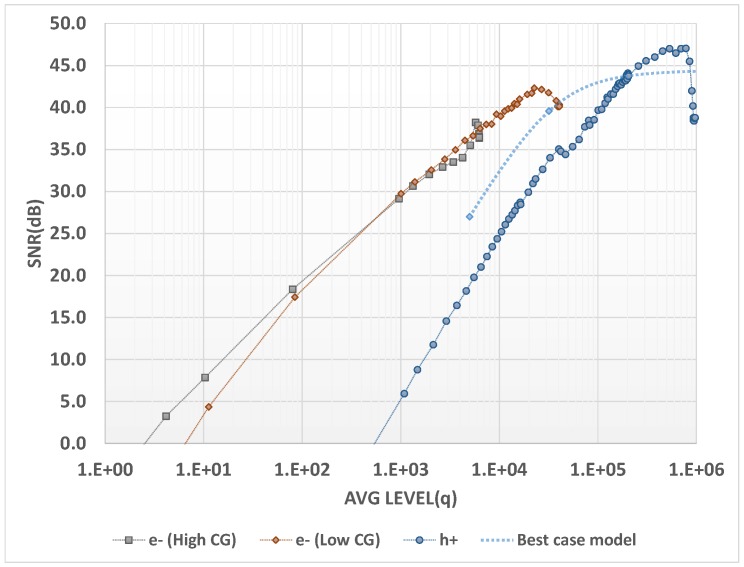
SNR versus charge.

In Figure 6, the measured SNR (signal-to-noise ratio) for this native HDR pixel is plotted for a 30 ms exposure. The total dark noise for the hole readout in this condition is 550 h+, with the main contributor being the dark current non-uniformity (DSNU, 515 h+). A theoretical SNR curve is computed by removing this DSNU contribution and considering 0.6% PRNU.

With HDR sensors, one of the major image quality criteria is the SNR value when the signal level is in transition between the low-light and high-light regions of operation. This SNR dip is usually dominated by photon shot noise because the amount of charges is substantially lower in the high-light image. Here, there is a continuity in the amount of charges and the SNR dip is purely the consequence of increased read noise.

At a transition point of, e.g., a 30 kh+ signal, the theoretical noise breakdown is almost equally shared among shot noise (173 h+), kTC noise (163 h+), ADC plus source follower noises (135 h+), and the ideal 0.6% PRNU (180 h+). Combining these contributors brings the total noise to 325 h+. This noise corresponds to 39 dB SNR at transition, which is the best performance that can be achieved with this pixel design. The actual SNR dip value measured on our sample at image transition is 32 dB, limited primarily by dark current non-uniformity and pixel response non-uniformity. Both of these metrics can be improved by process tuning.

On the hole SNR curve, a discontinuity is visible around 40 kh+. The main hypothesis for this artifact is uncomplete electron extraction when the photodiode is highly saturated.

### 5.2. Pixel Performance

The manufacturing process used for the sensor is a back-side illuminated (BSI) bulk process including capacitive deep trench isolation (CDTI), a color filter with a Bayer pattern, and a micro-lens. The pixel pitch is 3.2 μm. For this first implementation, a 600 × 650 image sensor test chip was produced comprising sub-arrays of different pixel variants (with 65 k pixels per variant).

The measured optical performance is presented in Table 3. The usable linear full well for electrons is 33 ke− in low CG mode and the usable linear full well for holes reaches 750 kh+. The ratio between the usable linear full well for holes and the electron noise floor in high CG mode yields a dynamic range of 116 dB. Clearly, the dark current for holes is the foremost image quality detractor. It is due to junction leakage at the transfer gate drain. No specific process optimizations have been made to contain this leakage on the measured samples. Fine tuning of this drain junction should enable a significant reduction of the dark current.

**Figure 7:**
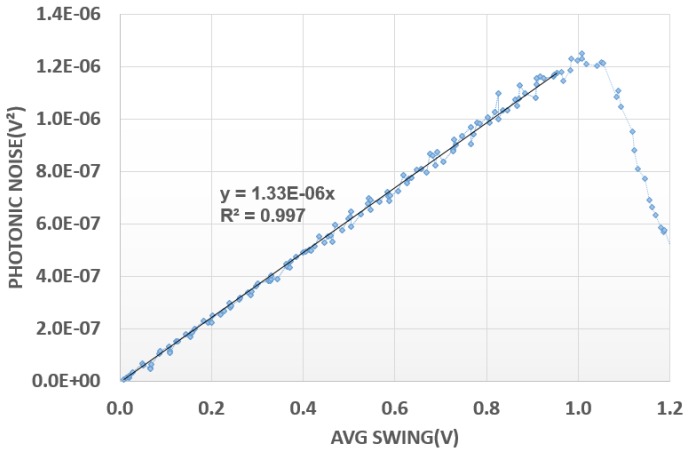
Hole photonic noise.

The measurement of the conversion factor for holes is an area of interest here. Using the photonic noise approach, the measured noise is in the low-millivolts region regardless of the signal and could be easily increased by any parasitic noise. As shown in Figure 7, this small noise is linearly dependent upon the square root of the signal and the value of 1.33 µV per hole is extracted from the curve.

**Figure 8:**
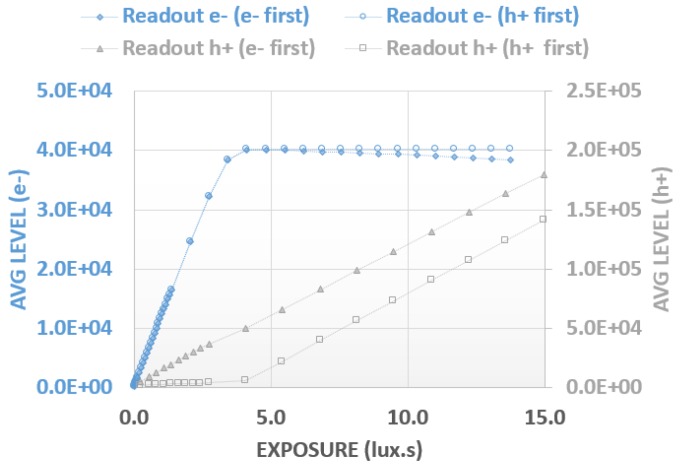
Linearity versus readout mode.

**Figure 9:**
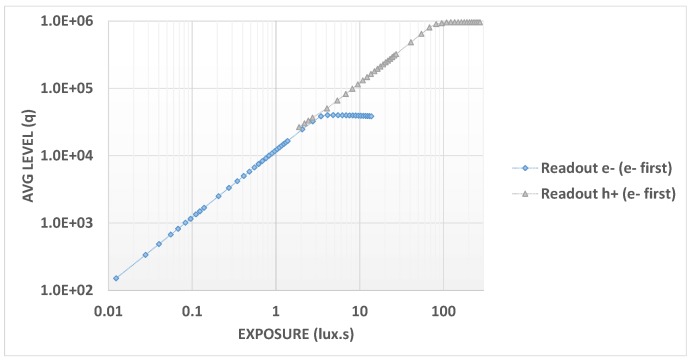
Linearity versus exposure.

Figure 8 shows electron and hole signal linearity with either hole-first or electron-first extraction. We see that in the first part of the curve, the electron signal is the same for both modes but starts to diverge after the full well is reached. For the hole signal, there is the expected flat response at the beginning for the hole-first mode whereas in the electron-first mode, this artifact is absent, giving a highly linear response. For the electron-first mode, the full linearity curve with a common X and Y axis for electrons and holes is plotted in Figure 9 in order to demonstrate the good overlap and the slope matching for both charge carriers.

**Figure 10:**
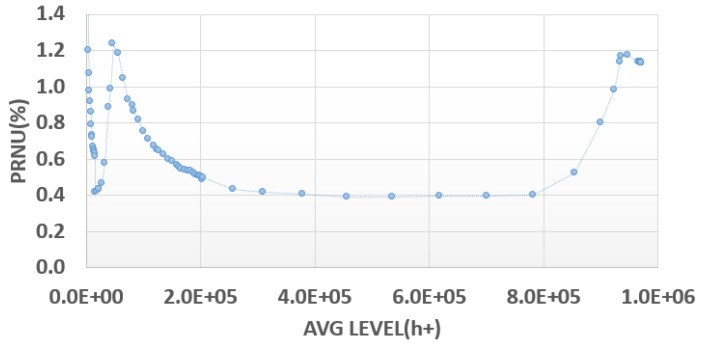
PRNU versus signal for holes.

The PRNU (Figure 10) exhibits very good behavior, with a value of 0.4% maintained across the majority of the signal range, validating the overall hole capture concept. A degradation of up to 1.5% is visible around the electron full well level and this is attributed to an incomplete reset of the photodiode as described previously.

**Figure 11:**
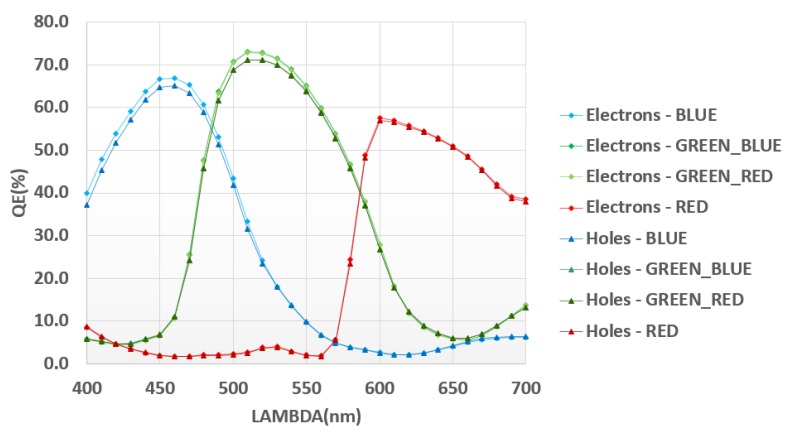
QE electrons and holes.

Quantum efficiency has been measured in both modes (Figure 11). Matching between both measurements over the full spectrum guarantees good HDR image reconstruction.

**Figure 12:**
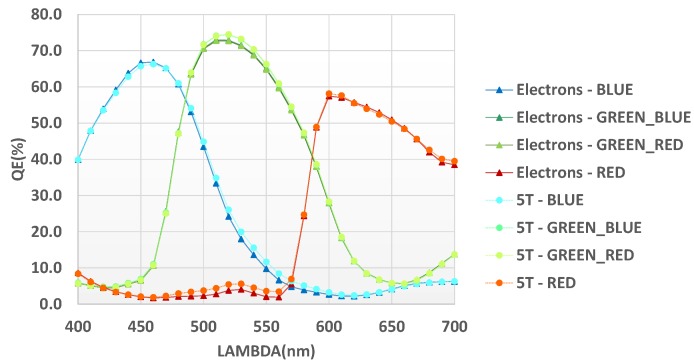
QE benchmark versus non-HDR pixel.

Finally, the interesting comparison of quantum efficiency versus a simple dual CG 5T 3.2 µm pixel measured on a wafer from the same lot (Figure 12) confirms the minor impact of pixel modifications on optical performance. A very limited reduction of green peak is shown. Also, the cross talk is notably improved thanks to the double CDTI wall between the diodes in the HDR pixel.

## 6. Conclusions

A native HDR pixel concept based on concurrent electron and hole collection has been demonstrated. It possesses a full well signal equivalent to 750 ke− with no compromise of sensitivity at low level. It operates with a single integration time, thus providing immunity to flicker or motion artifacts. Finally, the pixel operation is compatible with existing circuit design.

## Figures and Tables

**Table 1:** High dynamic range (HDR) approach comparison.

HDR Method	PRO	CONS	Flicker Immunity
Staggered images	HDR flexibility	Partial frame buffer Motion artifact	No
Chopped HDR	HDR flexibility	Frame buffer Motion artifact Frame length	Mitigation
Lin-log HDR	Single integration	Pixel FPN	No
Dual photodiode	No motion artifact	Pixel complexity Sensitivity loss	Yes
Lateral overflow	No motion artifact	High value capacitance integration	Yes
Complementary carrier collection	No motion artifact	P-type well separation needed	Yes

FPN: fixed pattern noise.

**Table 2:** Model input and associated temporal noise.

HDR Method	Parameter	Value
Common Values	Readout noise (SF + ADC)	150 µV
	Source follower gain	90%
	Voltage swing on column	800 mV
Dual photodiode	Diode sensitivity ratio	1 over 10
	Secondary diode full well	100 ke−
	Required capacitance	18 fF
	ktc + readout noise	83 e− (18 e− w/o ktc)
Lateral Overflow	Full well overflow storage	1 M e−
	Required capacitance	180 fF
	ktc + readout noise	316 e−
Complemetary carrier Collection	Full well holes	1 M h+
	Voltage swing on H node	2.5 V
	CDTI capacitance	64 fF
	ktc + readout noise	241 h+

SF: source follower; ADC: analog-to-digital conversion.

**Table 3:** Measured optical performance.

Performance	Unit	Value
High Conversion gain electrons	µV/e−	163
Low Conversion gain electrons	µV/e−	29.6
Conversion gain holes	µV/h+	1.33
Usable Full Well electrons	e−	33,000
Usable Full Well holes	h+	750,000
High Frequency PRNU electrons		0.55%
High Frequency PRNU holes		0.41%
Idark electrons	e−/s	45
Idark holes	h+/s	2300
Noise Floor in High CG Mode	e−	1.2
Dynamic range	dB	116
Green QE peak		73%

PRNU: pixel response non-uniformity; QE: quantum efficiency.
